# Limits of Usefulness of the X-Ray for the Diagnosis of Fractures

**Published:** 1899-03

**Authors:** 


### Limits of Usefulness of the X-Ray for the Diagnosis of Fractures.

On their first introduction it was thought that the X-rays would give absolute and immediate assurance of the presence of fractures in bones, and make it possible to dispense with the ripe clinical experience usually considered so necessary for exact diagnosis in the matter of certain fractures. Most of this promise has been fulfilled, but practical experience and the collation of expert opinions have shown that the X-rays have their limit of usefulness, and clinical experience is still of the greatest service in the diagnosis of these difficult conditions. The greatest care is necessary in the application of the X-rays in these cases, so that the position of the limb will not produce on the plate an impression of seeming deformity. Familiarity with skiagrams of the part is indispensable to the formation of an opinion in many cases as to the character of the displacement or solution of continuity that seems to be present in a given case. As a rule, practical expert knowledge of skiagraphy is necessary for reliable results in difficult cases, and even then the best results can only be secured by one who has a thorough clinical experience in the matter of fractures and dislocations, and who is able to decide intuitively, as it were, what is the position that will best bring out the deformity present. Under the circumstances the courts have done well to decide, in several cases where X-ray pictures were introduced as important evidence in damage suits, that as yet surgical opinion is not clear as to their absolute value in any given case, the angle at which they may be taken, the distance of the tube of origin of the rays and of the object to be skiagraphed from the sensitive plate, and, finally, the position of the part being factors in the production of appearances in the resultant skiagram that is impossible to properly value with absolute certainty. Recent series of skiagrams tend to confirm these opinions, and Professor Stimson has done well to illustrate this important point in an excellent series of skiagrams presented in his new work on "Fractures and Dislocations."*

We note among them a bimalleola Pott's fracture by inversion in a boy of fourteen years. The external malleolus is separated at the epiphyseal line, and the fracture shows very clearly. The fracture of the internal malleolus does not show in the skiagram, though recognized clinically by indubitable signs.

*A Treatise on Fractures and Dislocations. Lewis A. Stimson, M. A., M. D., Professor of Surgery in Cornell University Medical College. Lea Brothers & Co. Just Issued.

